# Policy challenges for the pediatric rheumatology workforce

**DOI:** 10.1186/1546-0096-10-5

**Published:** 2012-02-02

**Authors:** Carol B Lindsley

**Affiliations:** 1 Pediatric Rheumatology, University of Kansas Medical Center, 3901 Rainbow Blvd. Kansas City, KS 66160 913-588-5000, USA

## 

Dr. Henrickson presents a thoughtful three part analysis of current challenges to pediatric rheumatologists regarding barriers to access to care for children with rheumatic disease and to achieving optimal clinical outcomes [1-3]. He then presents some possible solutions. He has initiated an important conversation that has the potential to impact the future of our discipline.

In Part I he reviews the data that demonstrate the under-education of medical students and pediatric residents in musculoskeletal medicine in spite of the fact that children present to their primary care provider with frequent musculoskeletal complaints. Although over three-fourths of residency programs offer pediatric rheumatology (PR) rotations, the rate of participation is low due to a variety of factors including limited elective time, low interest, and lack of information about the subspecialty. He makes a plea for requirement that such a rotation be mandatory.

A mandatory requirement would put rheumatology in competition with other pediatric subspecialties, many of which have similar concerns regarding lack of exposure to their own subspecialty. Currently the Accreditation Council for Graduate Medical Education (ACGME) program requirements for general pediatrics do not require any specific non-intensive care subspecialty experience other than behavioral pediatrics and adolescent medicine. All other subspecialty requirements are institution- and program- specific. So, this type of mandatory requirement would be unprecedented and, in my opinion, unachievable in a general form. However, within individual institutions such a requirement can be achieved.

He discusses the role inadequate reimbursement or institutional support plays in access to care. The lack of parity of Medicaid-to-Medicare reimbursement in most states (all but 8) limits revenue for service and can be linked to poorer outcomes in Juvenile Rheumatoid Arthritis [4]. The disadvantageous Medicaid-to-Medicare fee index affects all of pediatrics but particularly subspecialties like pediatric rheumatology with limited procedures. In addition to wide-spread health care reform, possible solutions include increased efficiency in rheumatology practices, telemedicine to more rural areas, increased use of physician extenders and limiting low-risk referrals through improved physician education. All of these seem reasonable and potentially achievable. Telemedicine in particular merits increased study in rheumatology.

Part II deals with (1) barriers that limit patient access to self-management programs and multi-disciplinary team care as well as (2) insufficient workforce. Approximately 40% of the population with chronic disease reportedly does not receive adequate health care [5]. Of patients with chronic disease, musculoskeletal disease is the third most costly [6]. He discusses the Coleman model for chronic disease care and suggests that to implement it would require improved health care delivery system and support for a team approach. However, these changes are likely to occur slowly, as extensive restructuring of the healthcare finance system would be required.

With regards to the barrier of insufficient pediatric rheumatology workforce, studies point to the need for a 30% increase to meet the care needs [7]. Previous analyses suggest that although financial considerations are a factor for some, life-work balance and length of required training are also important [8]. He suggests that a clinically focused trainee could obtain all the required clinical experience in two years as well as complete a required scholarly project. Other recommendations include increase workforce diversification, flexible schedules for part-time practitioners and re-evaluation of the criteria for academic promotion.

A two-year training program would not be adequate for biomedical research activity or completion of a further graduate degree such as a MPH. It also would differentiate pediatric rheumatology from all other American Board of Pediatric subspecialties, all of which require three years of training. The adequate research time is particularly important in this subspecialty as 77% of trainees anticipate an academic career [7]. Of course, the percentage certainly might decrease if a two-year clinical track were also offered.

Part III. In this part Dr. Henrickson reviews the current pediatric global health priorities and anticipates the emergence of chronic conditions such as rheumatic disease as necessitating an increase in workforce in the future. Until the time when there are adequate pediatric rheumatologists or care extenders he makes a number of policy recommendations. These recommendations include, in part, structured musculoskeletal undergraduate modules, pediatric rheumatology world-wide outreach, increase in physician extender workforce training, regulatory reform of certifying organizations for these mid-level providers, use of telemedicine programs where feasible and on-site training opportunities for identified providers.

In summary, Dr. Henrickson has carefully analyzed workforce issues both in the US and internationally and made suggestions that should be seriously considered by the pediatric rheumatology community and implemented where feasible. The recognized need for an increased workforce over the next two decades can be successfully addressed and optimal rheumatologic outcomes in children achieved with the wide support of the pediatric rheumatology communilty.
